# Did the UK COVID-19 Lockdown Modify the Influence of Neighbourhood Disorder on Psychological Distress? Evidence From a Prospective Cohort Study

**DOI:** 10.3389/fpsyt.2021.702807

**Published:** 2021-06-21

**Authors:** Celine Teo, Chungah Kim, Andrew Nielsen, Thomas Young, Patricia O'Campo, Antony Chum

**Affiliations:** ^1^Department of Applied Health Sciences, Brock University, St. Catharines, ON, Canada; ^2^MAP Centre for Urban Health Solutions, Unity Health Toronto, Toronto, ON, Canada

**Keywords:** mental health, COVID-19, longitudinal study, public health, neighbourhood disorder, exogeneity, mobility, residential environment

## Abstract

**Background:** National lockdown in the UK during the COVID-19 pandemic severely restricted the mobility of residents and increased time spent in their residential neighbourhoods. This is a unique opportunity to understand how an exogenous factor that reduces mobility may influence the association between neighbourhood social environment and mental health. This study investigates whether the COVID-19 lockdown may modify the effect of neighbourhood disorder on psychological distress.

**Methods:** We tracked changes in psychological distress, using the UK household longitudinal survey across the pre-COVID and lockdown periods in 16,535 adults. Neighbourhood disorder was measured along two subscales: social stressors and property crime. Fixed-effects regression was used to evaluate whether the widespread reduction in mobility modifies the association between the subscales of neighbourhood disorder and psychological distress.

**Results:** The effect of neighbourhood social stressors on psychological distress was stronger in the lockdown period compared to the pre-COVID period. Compared to the pre-COVID period, the effect of being in neighbourhoods with the highest social stressors (compared to the lowest) on psychological distress increased by 20% during the lockdown. Meanwhile, the effect of neighbourhood property crime on mental health did not change during the lockdown.

**Conclusion:** The sudden loss of mobility as a result of COVID-19 lockdown is a unique opportunity to address the endogeneity problem as it relates to mobility and locational preferences in the study of neighbourhood effects on health. Vulnerable groups who have limited mobility are likely more sensitive to neighbourhood social stressors compared to the general population.

## Introduction

The World Health Organisation announced that the severe acute respiratory syndrome coronavirus 2 (SARS-CoV-2 or COVID-19) outbreak had become a global pandemic on March 11, 2020. Since then, as of April 2021, there have been 124 million cases and over 2.72 million deaths worldwide. In the UK, the government implemented a national lockdown (beginning on March 26, 2020) to help reduce the spread of COVID-19. The lockdown restricted the movements of residents from travelling outside their local area, and residents were urged from leaving their homes without a “reasonable excuse” (e.g., exercise, essential shopping, etc.) (1). These restrictions, along with mandatory face masking and limited social gatherings, were regularly enforced by the police which heavily restricted residents' mobility: an average of 1,423 fines per week were issued for restriction violations from March to July, 2020 (2). The UK COVID-19 lockdown presents a unique opportunity to study how mobility restrictions, which increase residents' exposure to their neighbourhood, can affect the relationship between neighbourhood disorder (i.e., perceived social and physical deterioration in their local area) and mental health. Since the lockdown introduced a rapid and unavoidable exogenous change in the duration of exposure to one's neighbourhood for the UK general population, we can exploit the opportunity to understand how the effects of neighbourhood disorder may shift when individuals experience a loss in their mobility.

Renewed interest in the influence of neighbourhoods on health has been the result of a rehabilitation of an ecological perspective in epidemiology, which recognises the embeddedness of individuals within larger social structures (3), where the residential neighbourhood represents one of the most intimate area-level organising structures to influence everyday life. Research in the area continues to build evidence on the health impacts of the physical dimensions of neighbourhoods (e.g., walkability (4) and access to community resources (5)) as well as the social dimension of neighbourhoods (e.g., sense of safety (6) and neighbourhood disorder (7)). Early efforts to measure neighbourhood social environments can be traced back to the Chicago school of urban sociology, and the idea that social disorganisation (or neighbourhood disorder) in neighbourhoods can undermine residents' well-being, and promote negative health outcomes (8). More recently, neighbourhood disorder is commonly detected through the presence of features such as littering, graffiti, vandalism, and property crime. These features, which convey to residents that they live in a neighbourhood with a high level of social and physical deterioration, generate persistent feelings of stress that contribute to increased allostatic load and hypervigilance (9). Prior literature suggests that neighbourhood disorder has negative impacts on residents' mental health. A systematic review found that eight out of nine studies on perceived neighbourhood disorder reported significant associations with depressive symptoms (10).

It is likely that neighbourhood disorder has differential effects on mental health across individuals with varying levels of exposure to the neighbourhood, given that studies on the effects of the neighbourhood environment provide evidence of a potential differential effect by the time spent in the neighbourhood (11–13). The effect of the neighbourhood environment on mental health has been found to be amplified among individuals with limited mobility, as seen in the older population (14). This magnified effect is theorised to be an artefact of the increase in exposure to the neighbourhood social environment, where proximal factors (i.e., vandalism, graffiti, loitering) become significantly more relevant compared to someone who is less confined to their neighbourhood boundaries (15, 16). Thus, we postulate that this effect would also be found in the relationship between neighbourhood disorder and mental health. However, the studies examining the effects of neighbourhood disorder on mental health may suffer from possible selection bias (i.e., an endogeneity problem), since potential confounders may influence the duration spent in the neighbourhood.

The endogeneity problem refers to the challenge that researchers face in disentangling neighbourhood effects from the effects associated with residents' decisions such as choice of neighbourhood or how much time is spent in the neighbourhood. It has been identified as a key limitation in prior literature examining neighbourhood effects on health (17–20). In the case of temporal-exposure endogeneity on a day-to-day level, individuals may choose to spend more or less time in their residential neighbourhood based on preferences, resources, perceptions of the neighbourhood, and level of mobility, among other factors.

Due to the forced restriction on mobility, the COVID-19 lockdown can be considered an exogenous change since residents' preferences no longer influence the duration of exposure to one's neighbourhood. This situation presents a unique opportunity to investigate whether an increase in exposure to neighbourhood disorder, without the influence of residents' choices and preferences, can modify the association between neighbourhood disorder and mental health. By exploiting the circumstances created by the COVID-19 lockdown that restricted mobility while minimising possible endogeneity, we aim to examine whether the lockdown amplified the effects of neighbourhood disorder on mental health.

## Methods

### Data

The *UK household longitudinal study* (UKHLS) is a large, nationally representative community-based survey following approximately 40,000 households since 2009 (21). Participants over the age of 16 who had responded in waves 8 or 9 were invited to participate in COVID-related web surveys that took place in the summer of 2020 (22). Household members who were deemed incapable to make an informed decision, living abroad, provided inaccurate postal addresses, and aged 15 and under in April 2020 were excluded from the survey. Our sample consisted of (*n* = 16,535) respondents who participated in Wave 9 (collected between January 2017 and June 2019) and at least 1 of the two COVID-19 web surveys collected between May and June 2020.

### Neighbourhood Disorder

Questions on neighbourhood disorder were asked in Wave 9, and respondents indicated on a scale of 1 (very common) to 4 (not very common), the extent of nine neighbourhood characteristics that include: home invasion, car theft, public intoxication, homelessness, graffiti, presence of trash, racial attacks, mugging, loitering, and vandalism. Since many of these factors are correlated, we followed prior studies in using data reduction techniques to extract distinct components to describe neighbourhood disorder (23, 24). Results of our principal component analysis extracted two distinct components with high item loadings (all at 0.66 or higher). Social stressors (the first subscale) consisted of five items including graffiti, presence of trash, loitering, vandalism, and public intoxication & homelessness. Property crime (the second subscale) was a function of two items, home invasion and car theft. Two variables (racial attacks and mugging) were omitted due to poor fit (i.e., defined as loading below 0.30) and were not used in the analysis. Cronbach's alpha reliability coefficient for internal consistency has a value of 0.85 for social stressors and 0.86 for property crime. The results of our principal component analysis are consistent with a seminal article on neighbourhood disorder (23). Our analysis extracted two components which are in agreement with the two dimensions described in the prior study: where the first dimension emphasised minor infractions that represent neighbourhood deterioration without a specific victim, and the second dimension are more serious crimes that threaten the safety of residents.

### General Health Questionnaire (GHQ-12)

The General Health Questionnaire (GHQ) is a validated survey tool used to measure participants' level of psychological distress over the past 2 weeks (25). The GHQ was included in Wave 9 and in the COVID-19 web surveys. It includes 12 questions about participants' mental health symptoms (e.g., trouble sleeping, problems concentrating, feeling under strain, loss of confidence, etc.) measured on a scale of 1 (not at all) to 4 (much more than usual). The UKHLS derived the scores into a single scale from 0 to 36, where a higher number on the scale represents a greater degree of psychological distress.

### Covariates

Our study utilises within-person estimators (individual fixed-effect), which adjust for the direct effect of time-invariant factors (i.e., ethnicity, immigrant status, gender, etc.), since each participant acts as their own control. To adjust for heterogeneity in the effect of the pandemic across population subgroups, we included the following variables as interaction terms with the COVID-19 period indicator: gender, adverse health conditions (i.e., physical or mental impairment that lasted for 12 months or longer), work from home status, and ethnicity (British White vs. other). We included an interaction term (i.e., lockdown effect^*^work from home status) to account for the potential differences in mobility across people who can vs. cannot work from home. A region-level fixed-effect was included to account for persistent regional differences where participants were categorised as a resident of the following twelve regions in the UK: North-East of England, North-West, Yorkshire and the Humber, East Midlands, West Midlands, East of England, London, South-East, South-West, Wales, Scotland, and Northern Ireland. The following time-varying confounders were included: employment status (employed, self-employed, unemployed, and furlough), natural log of age, weekly net household income (in thousand £), and the presence of COVID-19 symptoms (experienced COVID-related symptoms). To adjust for time trends, we included a time-fixed effect which is the number corresponding to the calendar month starting from Jan 2017 (time of our first interview).

### Statistical Analysis

A fixed-effects analysis was applied to this study to investigate the impact of COVID-19 on psychological distress across different levels of neighbourhood disorder (26). Since only the change in the outcome over the study period is being modelled, this approach controls for the impact of observed and unobserved time-invariant confounders. Change in GHQ was predicted by: (1) COVID-19 period indicator, (2) interactions between COVID-19 period indicator the two subscales of neighbourhood disorder (i.e., social stressors and property crime), (3) interactions of COVID-19 period by gender, adverse health conditions, work from home status, and ethnicity, (4) individual- and region-level fixed-effects, (5) underlying trend effect, and (6) time-variant control variables (e.g., change in employment status and income).

To deal with missing data, we analysed the full, incomplete dataset using maximum likelihood estimation (MLE). This method does not impute any data, instead it uses each case's available data to compute MLE based on the distributional properties of the statistical model. The likelihood is computed separately for those cases with missing data and those with complete data on all variables. These two likelihoods are maximised together to find the estimates. Prior studies provided evidence that MLE performed similarly to multiple imputation in its ability to provide unbiased parameter estimates and standard errors in empirical and simulation studies with missing data (27, 28).

We used a post-estimation method with the margins command in Stata 16.1 (Stata Corporation, College Station, Texas) to intuitively present the size and significance of the interaction effect on the outcome (29). We calculated the marginal effects of neighbourhood disorder, before and during lockdown, on the predicted GHQ, while holding all else at their means. We conducted first difference tests (separately for the pre- and during COVID periods) to examine whether there were significant differences in the predicted GHQ scores across individuals in neighbourhoods with different levels of disorder. We then carried out the second difference test, which investigated whether the marginal effects of neighbourhood disorder changed before and after the lockdown, while holding all else constant. In other words, the second difference shows whether the effects of neighbourhood disorder on mental health are contingent upon the implementation of the COVID-19 lockdown. All post-estimation tests were done separately for two subscales of neighbourhood disorder: social stressors and property crime.

## Results

Table 1 presents the sample characteristics of our study cohort at baseline along with the mean GHQ (and SD) scores at each level (where higher GHQ represents higher levels of psychological distress). Based on unadjusted analyses using Kruskal-Wallis non-parametric 1-way analysis of variance, we note that the following predictors are significantly associated with psychological distress in our sample: social stressors, property crime, gender, employment status, age, adverse health condition, and household income (see Table 1).

**Table 1:** Baseline characteristics of study cohort from UK Household Longitudinal Study, *n* = 16,535.

***N*** **=** **16,535**		
	**% (*****n*****)**	**Mean GHQ (SD)**
**Social stressors (ND1)**		
Lowest (Q1)	1,417	10.8 (5.11)
Medium-low (Q2)	4,636	10.8 (5.26)
Medium-high (Q3)	7,357	11.5 (5.53)
Highest (Q4)	3,125	12.9 (6.63)
Missing	0	n/a
*p*-value for difference in means	<0.001	
**Property crime (ND2)**		
Lowest (Q1)	869	11.0 (5.51)
Medium-low (Q2)	3,652	11.4 (5.42)
Medium-high (Q3)	5,211	11.0 (5.33)
Highest (Q4)	6,803	11.9 (6.02)
Missing	0	n/a
*p*-value for difference in means	<0.001	
**Gender**		
Men	7,005	10.5 (5.07)
Women	9,530	11.7 (5.70)
Missing	1	n/a
*p*-value for difference in means	<0.001	
**Employment status**		
Unemployed	5,748	11.0 (5.71)
Employed	8,403	11.2 (5.24)
Self-employed	1,249	10.7 (5.15)
Furloughed	n/a (furlough did not exist pre-COVID)	n/a
Missing	1,135	11.9 (6.24)
*p*-value for difference in means	<0.001	
**Age**		
<25	1,308	12.1 (6.11)
25–44	4,696	11.6 (5.77)
45–64	6,920	11.4 (5.59)
65+	3,609	9.67 (4.20)
Missing	2	13.5 (6.36)
p-value for difference in means	<0.001	
**Adverse health condition**		
Present	5,545	12.9 (6.51)
Absent	10,976	10.3 (4.63)
Missing	14	10.6 (3.99)
p-value for difference in means	<0.001	
**Household Income**		
Q1 (<549.55)	4,134	11.9 (6.23)
Q2 (549.55–821.92)	4,134	11.1 (5.49)
Q3 (821.92–1170.86)	4,133	10.9 (5.06)
Q4 (>1170.86)	4,134	10.7 (4.94)
Missing	n/a	-
*p*-value for difference in means	<0.001	

The main model from the fixed-effect analysis is found in Supplementary Table 1. Results of post-estimation analysis based on the estimates in the fixed-effect model are presented in Table 2 for the marginal effects on the predicted GHQ score before and during the COVID-19 lockdown period. The results of the first difference test (i.e., test of differences in the predicted GHQ across distinct levels of neighbourhood disorder) indicate that for both pre-COVID and lockdown periods, people reporting the highest level of neighbourhood disorder experienced the most psychological distress compared to those reporting lower levels of neighbourhood disorder (for both social stressors and property crime). We observed a consistently significant dose-response relationship for neighbourhood social stressors (Table 2) in both the pre-COVID and lockdown periods; however, the same effect was not observed for neighbourhood property crimes.

**Table 2:** Post-test estimation analysis from fixed-effect model predicting change in GHQ predicted by.

	**Pre-Covid**	**First-difference (difference in GHQ vs. Q4 in the pre-covid period)^**†**^**	**During-COVID**	**First-difference (difference in GHQ vs. Q4 during covid-19)^**†**^**	**Second-difference (difference in change over time compared to Q4)**
**Neighbourhood disorder subscale 1: Social Stressor**					
Lowest (Q1)	9.93^***^ (CI: 9.25–10.61)	12.35–9.93 = 2.42^***^ (CI: 0.92–3.91)	11.64^***^ (CI: 11.30–11.98)	14.55–11.64 = 2.90^***^ (CI: 1.37–4.44)	2.90–2.42 = 0.48^**^ (CI: 0.13–0.83)
Mid-Low (Q2)	10.32^***^ (CI: 9.84–10.80)	12.35–10.32 = 2.02^***^ (CI: 0.73–3.32)	12.11^***^ (CI: 11.97–12.25)	14.55–12.11 = 2.43^***^ (CI: 1.10–3.77)	2.43–2.02 = 0.41^*^ (CI: 0.08–0.73)
Mid-High (Q3)	11.28^***^ (CI: 11.10–11.45)	12.35–11.28 = 1.07^***^ (CI: 0.40–1.75)	13.09^***^ (CI: 12.54–13.65)	14.55–13.09 = 1.45^***^ (CI: 0.69–2.21)	1.45–1.07 = 0.37^*^ (CI: 0.04–0.71)
Highest (Q4)	12.35^***^ (CI: 11.53–13.18)	Reference group	14.55^***^ (CI: 13.30–15.79)	Reference group	Reference group
**Neighbourhood disorder subscale 2: Property crime**					
Lowest (Q1)	10.89^***^ (CI: 10.71–11.06)	11.31–10.89= 0.42^*^ (CI: 0.04–0.80)	12.66^***^ (CI: 12.37–12.95)	13.31–12.66 = 0.67^*^ (CI: 0.13–1.22)	0.67–0.42 = 0.25 (CI: −0.14–0.65)
Mid-Low (Q2)	10.64^***^ (CI: 10.40–10.88)	11.31–10.64= 0.66^**^ (CI: 0.25–1.08)	12.48^***^ (CI: 12.27–12.703)	13.31–12.48 = 0.85^**^ (CI: 0.28–1.43)	0.85–0.66 = 0.18 (CI: −0.21–0.59)
Mid-High (Q3)	10.17^***^ (CI: 9.62–10.72)	11.31–10.17= 1.14^**^ (CI: 0.42–1.85)	11.98^***^ (CI: 11.74–12.21)	13.34–11.98 = 1.36^**^ (CI: 0.54–2.17)	1.36–1.14 = 0.22 (CI: −0.19–0.63)
Highest (Q4)	11.31^***^ (CI: 11.07–11.55)	Reference group	13.34^***^ (CI: 12.65–14.02)	Reference group	Reference group

* *p < 0.05*,

** *p < 0.01*,

*** *p < 0.001*.

† *The derived values represent the first difference, which is the disparity between distinct levels of neighbourhood disorder compared to the reference group (Q4) for both pre- and during-COVID*.

The test of second difference shows that the effects of social stressors on psychological distress became stronger after the lockdown implementation. Specifically, we observed a difference of 2.42 between the Q1 (lowest stressor) and Q4 (highest stressor) in the pre-COVID period, compared to 2.90 between Q1 and Q4 in during the lockdown, which demonstrates that the gap between the Q1 and Q4 saw a 0.48 additional increase (second difference) between the pre-COVID and lockdown periods (95% CI 0.13–0.83). In addition, the size of increases in the GHQ score with the lockdown is proportional to the gaps in the levels of social stressors. Specifically, we found a difference of 0.48 comparing Q1–Q4 (95% CI 0.13–0.83); a difference of 0.40 (95% CI 0.08–0.73) comparing Q2–Q4; and a difference of 0.38 (95% CI 0.04–0.71) when comparing Q3–Q4. Meanwhile, the disparity across neighbourhoods with different levels of property crime did not change between the pre-COVID and the lockdown period (Table 2 column 6 - under property crime).

## Discussion

We found that the effect of neighbourhood social stressors on psychological distress was stronger in the lockdown period compared to the pre-COVID period. Compared to the pre-COVID period, the effect of being in a neighbourhood with the highest social stressors (compared to the lowest) on psychological distress increased by 20% in the lockdown period. Since the lockdown was an exogenous change that limited most residents' mobility to their local area, the additional distress for those who live in disadvantaged neighbourhoods may be attributed to effect-amplification through increased exposure. This is supported by the dose-response effect found in the second difference: psychological distress was proportionally associated with the level of social stressors in the pre-COVID and lockdown periods (i.e., more social stressors were associated with more distress), but these associations were stronger during the lockdown compared to the pre-COVID period.

Our modelling also shows that the lockdown did not amplify the effects of property crime on psychological distress. However, the lockdown may have reduced residents' fear of property crime since the neighbourhood may benefit from a safety in numbers effect described in environmental criminology literature (30) (i.e., residents' sense of safety is improved through the number of neighbours who are able to observe potential criminal behaviours). This view is also supported by a study that found a 60% reduction in property crimes in England and Wales during the first month of the UK national lockdown (31). The authors of the study attributed the decline in property crimes to having more “eyes on the street” as a result of the lockdown (i.e., neighbours act as surveillance which deters property crime). Therefore, it is not unexpected that the effects of property crimes were not amplified by the exogenous increase in exposure to one's neighbourhood. Meanwhile, certain components of social stressors related to physical decay (i.e., graffiti, vandalism, and trash on the streets) tend to persist and are unlikely to change over the relatively short study period. Unlike property crimes, there was no reduction in public order crimes (which include loitering and public intoxication) during the months of the web surveys (31). Thus, we are more confident that the change in association between social stressor and psychological distress reflects the lockdown condition rather than other influences such as “eyes on the street” or the reduction of public order crimes.

Previous studies have provided evidence that increases in duration of neighbourhood environmental exposure moderated the association between neighbourhood environment and health (11). For instance, a study reported that longer duration at home amplified the relationship between the residential food environments and fruit and vegetable intake (13). Likewise, another study confirmed the importance of the duration of time spent in the neighbourhood by showing that varying duration of exposure to the residential environments have differential effects on the association between residential neighbourhood walkability and walking (12). Our findings are consistent with these prior results in that increased time spent in one's residential neighbourhood can amplify neighbourhood effects; however, the prior studies were cross-sectional and time spent in the neighbourhood was an endogenous factor to the models (11–13). Our study is a unique contribution to the literature since we took advantage of the exogenous effect of the lockdown (i.e., individuals were forced to spend more time at home and/or their local area), which provides stronger evidence for causal inference that increased exposure to the neighbourhood can amplify neighbourhood effects on mental health status.

Our study has the following limitations: perceptions of neighbourhood disorder are self-reported and may not be objective. Alternative methods of measuring neighbourhood disorder, such as systematic social observation (32, 33), can arguably provide a more objective measure (because it is conducted by a third party). However, observations from non-residents of a neighbourhood may not necessarily reflect residents' lived reality, since there may be a mismatch of what defines the boundaries of the neighbourhood from the residents' vs. the external observer's perspective (34). Moreover, self-reported measures can be informed by a deeper knowledge of the neighbourhood through lived experience (35). Another limitation is that there may be other time-varying factors that could potentially confound the association between the COVID-effect and mental health in the model, such as a change in marital status over the study period. On the other hand, our models effectively use a time fixed-effect to adjust for time-variant macro-level changes; and an individual-level fixed-effect, which accounts for the influence of unobserved time-invariant confounders (e.g., marital status that has not changed over the study period). Moreover, since the mean study period is short (18 months) in the present paper, it is less likely to be susceptible to the effects of time-variant confounders. Lastly, our findings may not be generalizable to low- and middle-income countries, where the way people make use of their local neighbourhood and interact with neighbours is different.

Our study provides evidence that perceived neighbourhood social stressors become more important to residents with increased exposure. In addition, living in a neighbourhood with less social stressors has a protective effect on mental health over the course of a lockdown. Outside of the pandemic context, our research can inform future neighbourhood planning and interventions since it provides evidence that people with lower levels of mobility or spend more time in their neighbourhoods are the most affected: vulnerable subgroups include older adults, people with chronic illnesses and disabilities, and those who cannot afford or are unable to travel (13). Since they are more sensitive to their neighbourhood environments, their feedback should be prioritized when implementing systematic improvements to reduce neighbourhood social stressors. Best practices for area regeneration in the UK, aimed at improving neighbourhood resources and reducing stressors, recommended community participation at early stages of planning (36). Feedback from vulnerable subgroups should be prioritized, since these residents are most affected by their neighbourhood environment and may be less able to participate in the planning process. Future studies should evaluate the effectiveness of large-scale community-oriented neighbourhood regeneration, paying close attention to potential differential effects on individuals who have restricted or limited mobility, and increased exposure to their local neighbourhoods.

## Data Availability Statement

Publicly available datasets were analyzed in this study. This data can be found here: <https://www.understandingsociety.ac.uk/documentation/access-data>.

## Ethics Statement

The University of Essex Ethics Committee has approved all data collection on Understanding Society main study and innovation panel waves. Ethical review and approval for the use of secondary data was not required for current study.

## Author Contributions

CT and AC contributed to conception and design of the study. CT and CK performed the statistical analysis. CT, CK, AN, TY, and AC wrote the first draft of the manuscript. All authors contributed to manuscript revision, read, and approved the submitted version.

## Conflict of Interest

The authors declare that the research was conducted in the absence of any commercial or financial relationships that could be construed as a potential conflict of interest.
