# Supplementary material for: Did the UK COVID-19 Lockdown Modify the Influence of Neighbourhood Disorder on Psychological Distress? Evidence From a Prospective Cohort Study
Source: Front Psychiatry. 2021 Jun 21;12:702807. doi: 10.3389/fpsyt.2021.702807 (PMC8255607; doi:10.3389/fpsyt.2021.702807)
Supplement: Supplementary file 1 [file Table_1.DOCX]

Supplementary File

Table 1: Fixed-effects model predicting the effect of neighbourhood disorder on mental health (measured by GHQ) across participants before and during the COVID-19 lockdown

|  | Estimate | conf.low | conf.high | p.value |
| --- | --- | --- | --- | --- |
| **COVID effect** | 1.84 | 1.19 | 2.50 | <0.001 |
| **Interaction for social stressors (ND1) by COVID effect** | | | | |
| Lowest social stressors(Q1) | -0.65 | -1.04 | -0.26 | 0.001 |
| Medium-low social stressors (Q2) | -0.43 | -0.76 | -0.11 | 0.008 |
| Medium-high social stressors (Q3) | -0.42 | -0.76 | -0.07 | 0.016 |
| Highest social stressors (Q4) | Reference group | | | |
| **Interaction for property crime (ND2) by COVID effect** | | | | |
| Lowest property crime (Q1) | -0.40 | -0.82 | 0.01 | 0.059 |
| Medium-low property crime (Q2) | -0.27 | -0.69 | 0.14 | 0.198 |
| Medium-high property crime (Q3) | -0.21 | -0.62 | 0.20 | 0.310 |
| Highest property crime (Q4) | Reference group | | | |
| **Female by COVID effect** | 0.50 | 0.34 | 0.67 | <0.001 |
| **Adverse health conditions by COVID effect** | 0.70 | 0.52 | 0.87 | <0.001 |
| **Non-British white ethnicity by COVID effect** | 0.03 | -0.21 | 0.28 | 0.791 |
| **Work from home status by COVID effect** | -0.15 | -0.34 | 0.04 | 0.134 |
| **Employment status** |  |  |  |  |
| Unemployed | Reference group | | | |
| Employed | -2.88 | -0.70 | 0.12 | 0.172 |
| Self-Employed | -1.72 | -0.76 | 0.41 | 0.565 |
| Furlough | -0.46 | -1.07 | 0.14 | 0.135 |
| **Work from home** | 0.01 | -0.19 | 0.23 | 0.862 |
| **Household weekly earnings (in ￡1000)** | -0.05 | -0.14 | 0.03 | 0.235 |
| **Natural log of age** | -0.25 | -0.40 | -0.10 | 0.001 |
| **Experienced COVID symptoms** | 0.70 | 0.29 | 1.10 | <0.001 |
